# A 96-multiplex capillary electrophoresis screening platform for product based evolution of P450 BM3

**DOI:** 10.1038/s41598-019-52077-w

**Published:** 2019-10-29

**Authors:** Anna Gärtner, Anna Joëlle Ruff, Ulrich Schwaneberg

**Affiliations:** 1 Lehrstuhl für Biotechnologie, RWTH Aachen University, Worringerweg 3, 52074 Aachen, Germany; 2 DWI –Leibniz Institut für Interaktive Materialien, Forckenbeckstraße 50, 52074 Aachen, Germany

**Keywords:** Assay systems, Protein design

## Abstract

The main challenge that prevents a broader application of directed enzyme evolution is the lack of high-throughput screening systems with universal product analytics. Most directed evolution campaigns employ screening systems based on colorimetric or fluorogenic surrogate substrates or universal quantification methods such as nuclear magnetic resonance spectroscopy or mass spectrometry, which have not been advanced to achieve a high-throughput. Capillary electrophoresis with a universal UV-based product detection is a promising analytical tool to quantify product formation. Usage of a multiplex system allows the simultaneous measurement with 96 capillaries. A 96-multiplexed capillary electrophoresis (MP-CE) enables a throughput that is comparable to traditional direct evolution campaigns employing 96-well microtiter plates. Here, we report for the first time the usage of a MP-CE system for directed P450 BM3 evolution towards increased product formation (oxidation of alpha-isophorone to 4-hydroxy-isophorone; highest reached total turnover number after evolution campaign: 7120 mol_4-OH_ mol_P450_^−1^). The MP-CE platform was 3.5-fold more efficient in identification of beneficial variants than the standard cofactor (NADPH) screening system.

## Introduction

Monooxygenases are highly valuable enzymes from a synthetic perspective since they perform complex and specific oxidation reactions at ambient temperature using molecular oxygen. P450 monooxygenases convert a wide range of substrates like fatty acids, terpenes, steroids, prostaglandins, polyaromatic and heteroaromatic compounds, as well as a vast number of drugs, organic solvents, antibiotics, pesticides, carcinogens and toxins. Their high chemoselectivity makes them great candidates for biotechnological applications in synthesis. P450 BM3 from *Bacillus megaterium* has been employed in many successful protein evolution campaigns to enlarge the substrate scope or improve activity^1–4^, increase resistance in organic solvents^5^ and alter or enhance regio- and stereoselectivity^6–8^. Main limitation of P450 evolution is given by uncoupling, which describes the inefficient electron usage from the cofactor leading to reactive oxygen species (H_2_O_2_, ·OH) instead of product formation. While P450 BM3 shows a coupling efficiency of ~90% (C_14_–C_16_) with its natural substrates (medium and long chain saturated fatty acids)^9^, efficiencies of 0.1–71% were obtained with unnatural substrates like alkanes, cyclic alkenes and monosubstituted benzenes^1,2,4^. In addition to low product yields, the resulting reactive oxygen species are toxic to the catalyst and lead to inhibition of P450s during the course of reaction^10^. Therefore, the application of product specific screening systems for P450s plays a key role in the success of directed evolution campaigns.

Product based colorimetric and fluorescent screening systems have been developed in case of P450s for the detection of hydroquinones^11^, epoxides^12^, phenols^13,14^ and indigo^15^ as well as for enantioselectivity^16^. Alternatives with low throughput are analytical methods like high performance liquid chromatography (HPLC), gas chromatography (GC) or mass spectrometry (MS)^17–19^. Commonly employed alternatives with no direct product read out are cofactor based screening systems such as the NADPH depletion assay for dehydrogenases or P450s (measured absorbance at 340 nm)^20–23^. In case of P450s and usage of non-natural substrates, a main limitation of the NADPH screening systems is that cofactor usage is only partially linked to the target product formation. Determined activities do not only account for uncoupling and the target product, but also include formation of side products. The latter leads to overestimation of the monitored activity and target product formation for non-natural substrates^24^. Therefore, selection of robust variants for synthetic applications is often not possible with the NADPH screening system. That is why great efforts have been invested in colorimetric and fluorogenic screening systems. Universal product detection systems with high throughput would give a great benefit to the field of directed evolution.

One novel and promising analytical screening tool for directed evolution campaigns is capillary electrophoresis (CE) which can provide highly efficient separations with the possibility to quantify a broad range of compounds (e.g. nucleic acids, peptides, lipids, carbohydrates, metabolites and small molecules)^25,26^. CE has become a routine method in a number of industries including pharmaceutical analysis, forensic determinations and clinical analysis^27^ due to its broad applicability in separation of ionic compounds, neutral molecules, small molecules as well as biomacromolecules^28^. Thereby, the separation performance is tunable through variation of the selected background electrolyte e.g., with surfactants (micellar electrokinetic chromatography; MEKC)^29^, microemulsions (microemulsion electrokinetic chromatography; MEEKC)^30^, the use of non-aqueous solvents^31^ or chiral additives^32^. Principal advantages of CE are often low reagent costs, minimal sample requirements, and the possibility of multiplexing, which allows the simultaneous measurement through several capillaries within a single run and a single instrument^33,34^. Multi-capillary instrumentation in 96-well format (MP-CE), which was introduced in the market in 1991, was mainly employed for DNA analytics^35^. Its deployment within the Human Genome Project dramatically increased analytical speed and sample throughput of the initiative^36^. So far, multichannel systems were employed to perform high throughput screening of genetic mutations^37^ and clinical samples, in metabolomic and metabonomic studies^38^, of compound libraries to find potential and selective kinase inhibitors^39^ and of enzymatic activity under different reaction conditions^40^. To our knowledge, MP-CE has not been utilized for any protein evolution campaign, yet.

We herein present the first application of 96-channel-MP-CE for the high throughput screening of P450 BM3 libraries and a comparison to the NADPH screening system. The oxidation of alpha-isophorone was selected as target reaction due to the importance of 4-hydroxy-isophorone and keto-isophorone as intermediates for vitamin E synthesis^18,41^. P450 BM3 wild type (WT) was already successfully applied in an industrial demonstration process for 4-hydroxy-isophorone production by DSM Innovative Synthesis B.V. (Geleen, The Netherlands) reaching product concentrations of more than 6 g L^−1^ at a 100 L scale^41^. However, P450 BM3 WT suffered from self-deactivation with a high uncoupling of ~70% (experimentally determined in this publication). The specific detection of 4-hydroxy-isophorone and keto-isophorone is challenging since colorimetric and fluorimetric screening systems have not been reported. A 96-MP-CE platform was employed as product based screening systems for screening of P450 BM3 libraries towards improved activity for 4-hydroxy-isophorone production. Additionally, a comparison of the MP-CE to the NADPH screening systems was performed to benchmark the MP-CE system.

## Results

**Figure 1:**
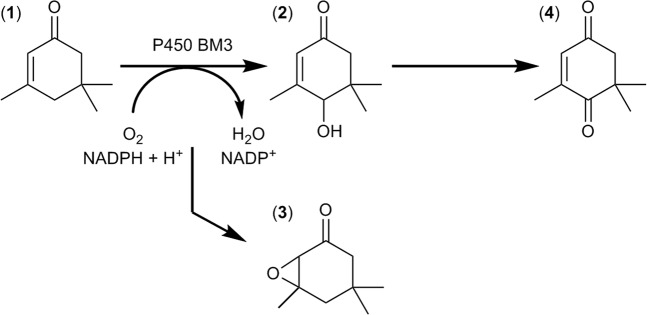
Reaction scheme of alpha-isophorone (1) conversion by P450 BM3. As main product 4-hydroxy-isophorone (2) is formed by the P450 BM3 WT. Potential enzymatic side products are isophorone oxide (3), the double oxidation product keto-isophorone (4) and several hydroxy-methyl products (not shown).

Multiplexed capillary electrophoresis systems (MP-CE) allow the simultaneous detection and quantification of a broad range of chemical compounds making them an excellent new tool for estimation of productivity and selectivity within directed evolution campaigns. After establishment of conditions for screening with a MP-CE platform, two site-saturation mutagenesis (SSM) libraries of P450 BM3 were screened towards increased activity for the oxidation of alpha-isophorone to 4-hydroxy-isophorone (Fig. 1). In the subsequent paragraph, the application of the MP-CE system for enzyme evolution is compared to the frequently used NADPH depletion assay in terms of performance in identification of P450 BM3 variants with increased 4-hydroxy-isophorone formation. The “best” performing variant from each of the two screening platforms was selected for further detailed characterization.

### Multiplexed capillary electrophoresis screening platform for identification of isophorone compounds

#### Establishment of the MP-CE screening platform

**Figure 2:**
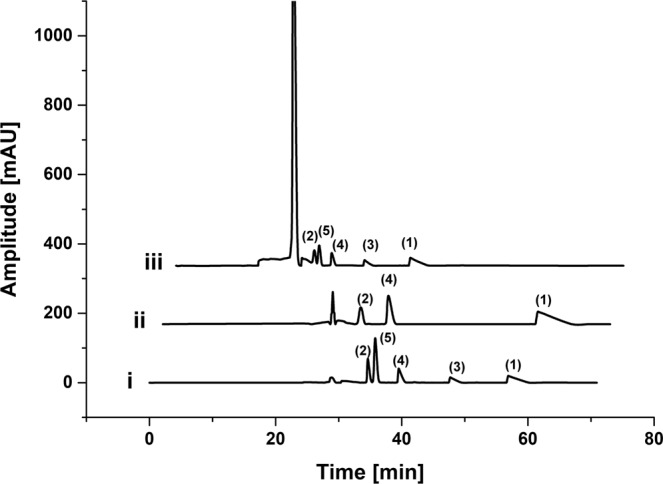
MP-CE electropherograms of isophorone standards. Isophorone derivatives ((1) alpha-isophorone, (2) 4-hydroxy-isophorone, (3) isophorone oxide and (4) keto-isophorone) and the internal standard (5) benzyl alcohol (5 mM each) were separated with different voltages and detected at different wavelengths (i) 4.5 kV, 214 nm; (ii) 4.5 kV, 254 nm; (iii) 8 kV, 214 nm to find suitable screening parameters.

The simultaneous detection of multiple compounds using analytical tools demands a sufficient peak resolution. Several buffer additives (alpha- and beta-cyclodextrin, urea, 18-crown-6-ether, SDS) were tested in varied concentrations (alone and in combinations; Supplementary Fig. S1) to achieve separation of four neutral isophorone derivatives ((1) alpha-isophorone, (2) 4-hydroxy-isophorone, (3) isophorone oxide, (4) keto-isophorone) and an internal standard ((5) benzyl alcohol) via multiplexed capillary electrophoresis (MP-CE). Usage of a micellar buffer system (30 mM SDS in a 15 mM sodium phosphate buffer, pH 7.45) provided suitable separation results (Fig. 2, lane i) and was henceforth used in subsequent experiments. UV detection was performed at 214 nm as detection at 254 nm was not feasible due to lack of absorbance of isophorone oxide (3) (Fig. 2, lane ii). Two different current voltages of 4.5 kV and 8 kV (Fig. 2, lane i and lane iii) were tested and both showed sufficient resolution of the isophorone derivatives (Table S1). However, usage of 4.5 kV resulted in higher peak resolution for the in close proximity eluting 4-hydroxy-isophorone and benzyl alcohol (1.59 vs. 1.11) while usage of 8 kV has the advantage of shorter running time (40 min vs. 60 min). In the following experiments 4.5 kV were used as separation current and standard curves were generated. Lower detection limits of 0.125 mM for alpha-isophorone, 4-hydroxy-isophorone and keto-isophorone and 0.5 mM for isophorone oxide (Supplementary Fig. S2) were observed with a linear behavior per isophorone derivate to at least 20 mM.

To ensure P450 BM3 product formation above the lower detection limit, NADPH regeneration was initiated by supply of glucose dehydrogenase (4 U mL^−1^) and glucose (60 mM) similar as published by Weingartner *et al*.^11^. Different NADPH regeneration times (3–18 hours) were investigated and 18 hours was identified as most suitable incubation interval for subsequent screening towards 4-hydroxy-isophorone formation. The MP-CE system showed a standard deviation of 12.2% ± 1.8% when quantifying the product formation of 4-hydroxy-isophorone by 90 P450 BM3 WT variants on one microtiter plate (MTP) (Supplementary Fig. S3).

### Multiplex capillary electrophoresis screening assay for P450 BM3 library screening

#### NADPH depletion assay vs. MP-CE screening

**Figure 3:**
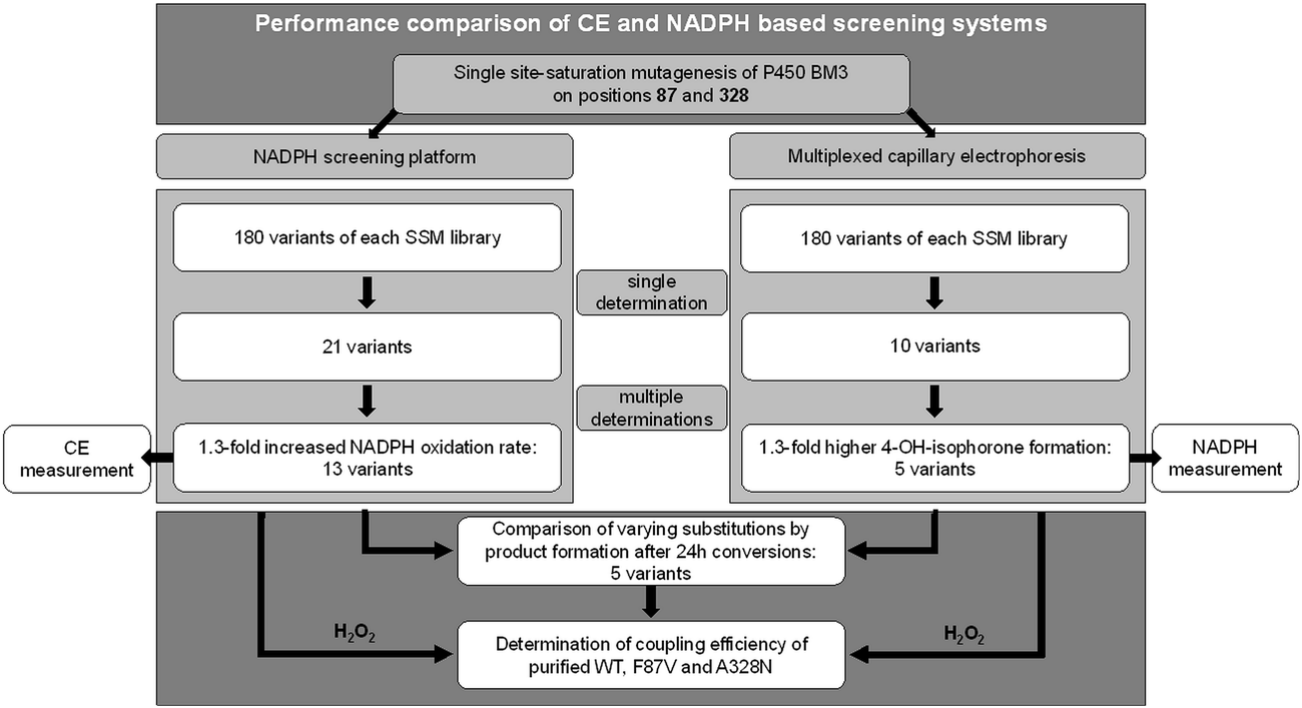
Flow chart of screening approach and variant characterization. Multiplexed capillary electrophoresis (MP-CE) was used as platform for screening of two P450 BM3 site-saturation mutagenesis libraries at positions F87 and A328 and compared to the frequently used NADPH depletion assay. A MP-CE and a NADPH based selection of variants was made and selected variants rescreened in multiplets in the corresponding platform. Variants with ≥1.3-fold increase in performance were selected and product formation of all identified substitutions compared to the WT in substrate conversion experiments. For a holistic view, rescreening was as well performed with the opposite platform. The two variants performing “best” in the respective platform (MP-CE: F87V, NADPH: A328N) were purified and their coupling efficiencies and initial oxidation rates determined.

**Figure 4:**
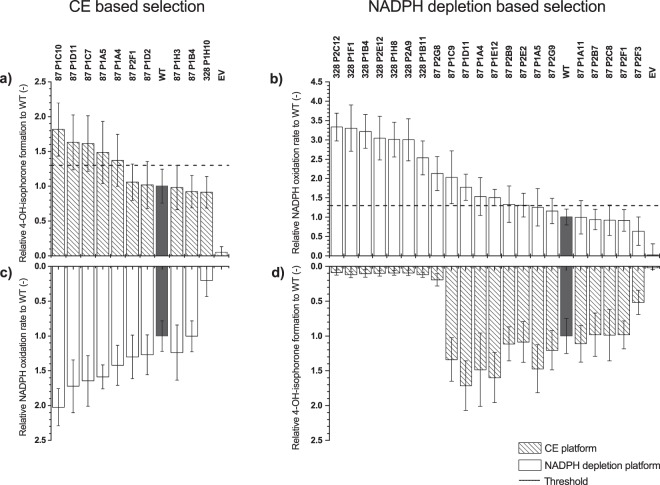
Rescreening of variants from MP-CE based selection, (**a,c**), and NADPH depletion based selection (**b,d**). Identified P450 BM3 variants from both screening methods were measured in triplicates and compared to the P450 BM3 WT (grey column) and EV (negative control). Variants which showed an increase of factor ≥1.3 (2.5-fold standard deviation, dashed line) in 4-hydroxy-isophorone formation (dashed columns) within the CE based selection (**a**) or NADPH oxidation rate (white columns) within the NADPH depletion based selection (**b**) were selected for further characterization. For a full picture, variants from the CE based selection (**c**) and NADPH depletion based selection (**d)** were screened with the opposite platform, however, results not considered for selection. (measurements: n = 4, mean ± s.e.; in biologically independent experiments) Substitutions of variants see Supplementary Table S2.

Two site-saturation mutagenesis libraries (SSM) at positions F87 and A328, SSM87 and SSM328, were chosen for the evaluation of the applicability of the MP-CE screening system for evolution campaigns (Fig. 3). P450 BM3 wild-type (WT) was chosen as starting variant and both positions, F87 and A328, separately saturated to all 20 canonical amino acids. Both positions are well known from several engineering campaigns of P450 BM3^17,42^. To ensure coverage of the full natural diversity (theoretical calculated >95%)^43^, 180 P450 BM3 variants of each library were screened with MP-CE (Supplementary Fig. S4). Subsequently, the comparison with the NADPH depletion assay^20^ (Supplementary Fig. S5), which is frequently used as indicator for initial variant activity in evolution campaigns, was undertaken. Variants with at least 1.4-fold higher 4-hydroxy-isophorone formation compared to the WT enzyme were considered as improved within the MP-CE based screening (10 variants); P450 BM3 variants with at least 1.4-fold increased NADPH oxidation rates were considered activity improved within the NADPH depletion based system (21 variants). Selected variants were measured again in triplicates (to minimize artefact selection) with the respective method. The threshold for selection was set to 1.3-fold increase to WT for both platforms. From the 10 variants from the MP-CE based selection, 5 variants (50.0%) showed improved 4-hydroxy-isophorone formation (Fig. 4a). All 5 variants were from SSM87 and contained the amino acid substitution phenylalanine to valine (Supplementary Table S2). From the 19 variants from the NADPH depletion based selection, 14 variants (73.7%) showed higher NADPH oxidation rates than P450 BM3 WT (Fig. 4b). Sequencing of these 14 variants (Supplementary Table S2) revealed that 4 of 7 variants from SSM87 contained valine as substitution, one variant threonine, one proline and one no substitution, respectively. From the 7 variants identified from the SSM library at position A328, one variant showed leucine as substitution and the remaining 6 variants asparagine. Identified P450 BM3 variants with different substitutions (F87V, F87P, F87T, A328N, and A328L) were selected for further product characterization (subsequent chapter).

P450 BM3 variants from the MP-CE based selection were analyzed with the NADPH screening systems and vice versa in order to compare both screening platforms. The NADPH oxidation rates from the MP-CE based selection (Fig. 4c) correlated with increased 4-hydroxy-isophorone formation (Fig. 4a). Within the NADPH depletion based selection, no parallelism between 4-hydroxy-isophorone formation and NADPH oxidation rates could be identified. Notably reduced 4-hydroxy-isophorone formation was observed especially for variants from SSM328 (9–12% of WT level; Fig. 4d) in the MP-CE system while exhibiting the highest NADPH oxidation rates with 2.5–3.2-fold increase to WT (Fig. 4b). Findings indicate that both platforms give different insights into the catalytic behavior of variants.

### Characterization of identified P450 BM3 variants

**Figure 5:**
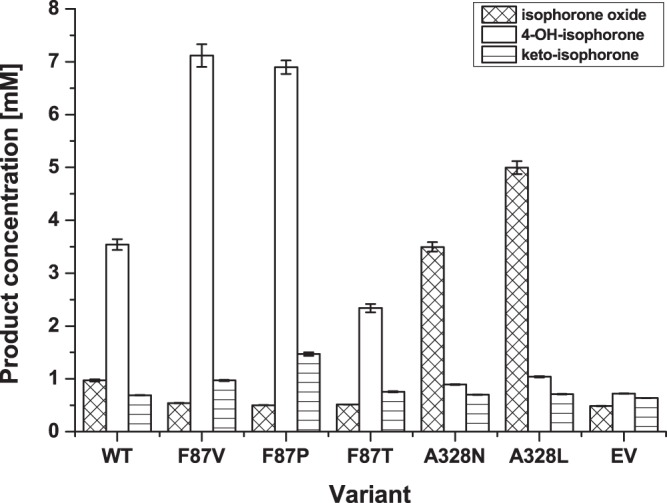
Product formation by variants identified via MP-CE and NADPH depletion based screening systems. Product formation after 24 h conversion of alpha-isophorone was quantified by GC-FID. Variant F87V was identified by MP-CE and NADPH depletion screening, and variants F87T, F87P, A328N and A328L by NADPH depletion assay. Conversion was performed in triplicates with normalized P450 content using lysates and a NADPH regeneration system. Lysate of cells expressing the empty vector (EV) not carrying a P450 BM3 gene was taken as negative control. (measurements: n = 3, mean ± s.e.; in biologically independent experiments).

Identified substitutions from both screening systems (F87V, F87P, F87T, A328N, A328L) were chosen to perform conversion of alpha-isophorone with respective variants for 24 h in presence of a cofactor regeneration system to quantify product formation with gas chromatography using flame ionization detection (GC-FID) (Fig. 5).

P450 BM3 WT showed a formation of 3.54 mM µM_P__450_^−1^ of the target product 4-hydroxy-isophorone with 84% regioselectivity.

P450 BM3 F87V, which was the only variant identified within the MP-CE screening, showed a 2.01-fold (7.12 mM µM_P__450_^−1^) improved 4-hydroxy-isophorone formation with 94% regioselectivity. One of the 5 substitutions identified within the NADPH based selection was F87V, which was as well selected in the MP-CE screening with previous mentioned improvements. P450 BM3 F87P showed as well an increased 4-hydroxy-isophorone formation (1.95-fold to WT), however, exhibited a strong tendency to incorrect folding as observed in recorded CO-difference spectra (Supplementary Fig. S6). Decreased 4-hydroxy-isophorone formation (2.34 mM µM_P__450_^−1^) was observed with variant F87T. Both latter variants, F87P and F87T, exhibited relaxed regioselectivity, which led to further side products (GC chromatograms, Supplementary Fig. S7) of unknown character. Two different variants were identified from SSM328, A328N and A328L, which showed the highest NADPH oxidation rates within the rescreening (~2.4–3.2-fold increased to WT, Fig. 4b). Product profiles of both variants were shifted strongly towards formation of the undesired side product isophorone oxide up to 3.6–5.2-fold (3.50–5.00 mM µM_P__450_^−1^) whilst formation of 4-hydroxy-isophorone decreased to 0.25-fold (0.89 mM µM_P__450_^−1^) and 0.29-fold (1.04 mM µM_P__450_^−1^) for A328N and A328L, respectively.

**Figure 6:**
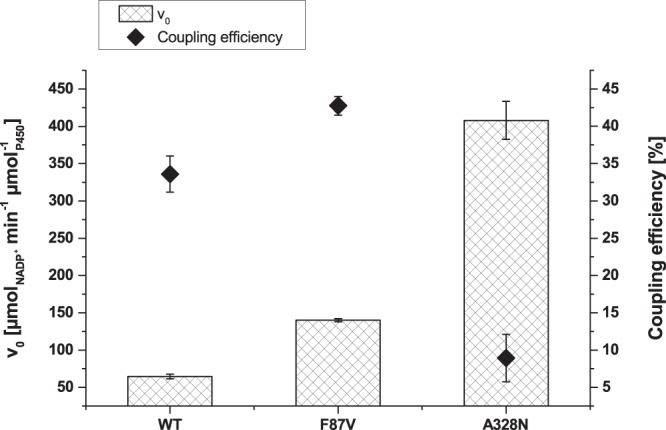
Performance characteristics of purified P450 BM3 WT and variants F87V and A328N. Initial NADPH oxidation rates (columns) as a measure for activity and coupling efficiencies (♦) of the variants performing “best” in the MP-CE based screening platform (F87V) and the NADPH based screening platform (A328N), respectively, were determined for characterization in triplicates. (measurements: n = 2, mean ± s.e.; in technically independent experiments).

The variant, which displayed the highest improvements in 4-hydroxy-isophorone formation in the MP-CE based selection (F87V), and the variant with highest NADPH oxidation rate in the NADPH depletion based selection (A328N) (Supplementary Table S2) were chosen for purification and detailed characterization. Initial NADPH oxidation rates of A328N and F87V were 6.3- and 2.2-fold improved (Fig. 6), respectively, while coupling efficiency increased 1.27-fold for F87V (42.75%) compared to the WT (33.59%) and decreased 0.27-fold for A328N (8.92%).

These results clearly demonstrate a high gap between NADPH consumption and product formation of variant A328N while variant F87V shows a balanced increase in both characteristics.

## Discussion

Cytochrome P450 monooxygenases form an important superfamily that catalyzes oxygenation reactions, mainly hydroxylation and epoxidation, of non-functionalized hydrocarbons. Enzymatic oxidations certainly expand and enrich our synthetic capabilities in organic chemistry, especially if they are performed in a highly chemo- and stereospecific manner as observed with many P450 monooxygenases. One example of industrial P450 catalysis applying P450 BM3 WT is the successful production of a vitamin E synthesis intermediate, 4-hydroxy-isophorone, through an enzymatic para-hydroxylation of alpha-isophorone^41^. Product concentrations of more than 6 g L^−1^ at a 100 L scale were achieved in latter demonstration case, however, P450 BM3 WT performance was restricted by “self-deactivation” resulting in limited total turnover number (TTN: 18 000 mol_4-OH_ mol_P450_^−1^). While P450 BM3 features excellent prerequisites for industrial application, it is likely the coupling efficiency limiting its productivity and preventing its broad industrial application. At present time, high catalyst loads are necessary to obtain industrially sufficient yields which is from an economic perspective only feasible for high-value products (see data fact sheets of the EU ROBOX project on oxygenation catalysis: https://h2020robox.eu/wp-content/uploads/2017/02/D5.1-%E2%80%93-Completion-of-benchmarking-protocol-and-flowsheets-established-for-the-case-studies.pdf). Over the past decade, impressive advances have been achieved via protein engineering in respect to activity, stability, and specificity of P450s^44,45^. A substrate independent screening tool to monitor oxygenase and dehydrogenase activity is the measurement of the depletion or formation of their cofactor NADPH or NADH, respectively^46^. In case of oxygenases, often non- or poorly transferable results are obtained as side effect of increased uncoupling if non-natural substrates are used in directed evolution campaigns. Therefore, direct read out of the product, which enables to identify P450 variants with high coupling efficiencies, is of utmost interest to generate industrially applicable P450 biocatalysts. Detection of 4-hydroxy-isophorone during high-throughput screening is challenging as fluorescent properties and a specific absorbance peak in the UV/vis-area are missing. In addition, establishment of enzymatically and chemically coupled detection systems is hampered for instance by steric hindrance caused by three neighbouring methyl groups^18^, and by lack of simple, water tolerable derivatization reactions specific for secondary alcohols (formation of hydroxy-methyl side product by P450 BM3 variants possible^47^). So far, mostly analytical methods with medium throughput like HPLC have been used to screen P450 libraries towards improved 4-hydroxy-isophorone formation^18^.

For the first time multiplexed capillary electrophoresis was applied as a general product based screening method for an enzyme evolution campaign. The monooxygenase P450 BM3 was chosen as biocatalyst. The MP-CE platform allowed the direct detection of the target product 4-hydroxy-isophorone in up to 96-channels, and the simultaneous detection of the substrate alpha-isophorone as well as two biocatalysis side products (keto-isophorone, isophorone oxide) in a micellar buffer system supplying 30 mM SDS into 15 mM sodium phosphate, pH 7.45 (for further discussion on separation buffer see Supplementary). A broad linear detection range (0.125 mM to at least 20 mM), sufficient resolution (>1.59) and a standard deviation of 12.2% (measuring the 4-hydroxy-isophorone formation of 90 replicates of P450 BM3 WT) enabled reliable screening for P450 BM3 variants with improved product titers and improved coupling efficiency. As previously shown by Wong *et al*.^13,^ screening systems with a standard deviation around 12% are sufficient for successful directed evolution campaigns. A NADPH regeneration system with glucose and glucose dehydrogenase was applied to minimize use of the expensive cofactor NADPH and achieve product formation within the linear detection range. Sufficient throughput was achieved with the possibility to screen a theoretical number of 2160 variants/day (60 min separation at 4.5 kV for one MTP with 90 variants). Throughput of the MP-CE system could even be improved by increase of the separation voltage (Fig. 2), however, not without slight reduction of resolution of the closely eluting 4-hydroxy-isophorone and the internal standard benzyl alcohol (Supplementary Table S1). In comparison to single channel analytical tools, the MP-CE platform is more tolerant to small variations between runs caused by differences in e.g., pH, additive concentration, temperature. The multiple channel system allows parallel measurement of controls for each run, thus, latter differences apply for all samples on one MTP and relation between controls and variants should stay unaffected.

The MP-CE platform was employed to screen two SSM libraries at positions F87 and A328 with 180 variants each toward increased 4-hydroxy-isophorone formation. Both amino acid positions are reported to strongly influence regioselectivity and substrate specificity of P450 BM3^17,42^. Only one promising substitution, F87V, was identified from both SSM libraries after rescreening (Fig. 4a, Supplementary Table S2) and indeed, variant F87V showed improvements in NADPH oxidation rate, regioselectivity, and coupling efficiency resulting into a 2.01-fold increased 4-hydroxy-isophorone formation (Figs 5 and 6). An incredible accuracy of 100% for truly improved variants (5 out of 5 variants identified as F87V) was achieved after performing rescreening with the MP-CE platform (Supplementary Table S2). From the selection after initial screening considerable 50% (5 out of 10 variants) were the P450 BM3 variant F87V.

In contrast, screening of both libraries with the commonly employed NADPH cofactor depletion platform resulted into 5 substitutions (F87V, F87T, F87P, A328N and A328L; Supplementary Table S2) with potential beneficial effects. However, the most predominant and promising (highest NADPH oxidation rates) variant A328N (Fig. 4b) showed 0.27-fold reduced coupling efficiency compared to the WT enzyme (Fig. 6) and a shift of regioselectivity towards epoxide formation (Fig. 5). Identification and characterization of A328N clearly demonstrates the discrepancy between high NADPH oxidation rates, target product formation, and effective co-factor usage. The latter findings associated to variant P450 BM3 A328N underline how NADPH oxidation rate based screening campaigns can mislead toward selection of false positive variants with mainly side product formation. Nevertheless, NADPH depletion screening enabled the identification of the truly improved and robust variant F87V (28.6%, 4 out of 14 variants after rescreening) (Supplementary Table S2).

Both screening platforms, MP-CE and NADPH depletion, enabled to identify the most beneficial variant P450 BM3 F87V. Employing MP-CE allowed variant selection with a 3.5-fold increased accuracy (exclusive identification of variant F87V after rescreening; 100%) in comparison to the NADPH depletion platform (28.6% correctly identified). In addition, efforts for subsequent variant characterization were strongly reduced as false positive hits are excluded at early stages of screening (Supplementary Fig. S8). Platform comparison clearly demonstrated the strong benefits of product based screening methods in directed evolution (for detailed comparison of key factors of HTS systems see Supplementary Table S3).

Capillary electrophoresis has a broad acceptance in bioanalysis and is employed in several bioassays^26,28,48^. The developed MP-CE platform demonstrated the first application of 96-channel-multiplex capillary electrophoresis as high-throughput screening platform for a monooxygenase evolution campaign. In conclusion, the MP-CE platform allowed quantitative detection of multiple compounds, which led to a reliable product based variant selection. The MP-CE tool fulfilled the prerequisites for an effective biocatalyst screening platform. Together with the number of options available for adaption of capillary electrophoresis for analytical problems^49^, MP-CE will be decisive for the technology breakthrough in directed evolution.

## Online Methods

All used chemicals were purchased from Sigma-Aldrich (Steinheim, Germany), Roth (Karlsruhe, Germany) or Merck (Darmstadt, Germany) if not stated otherwise. Restriction enzyme *Dpn*I and dNTPs were purchased from New England Biolabs (Frankfurt a. M., Germany). Salt-free oligonucleotides (HPSF purity) were obtained from Eurofins Genomics (Ebersberg, Germany). Glucose dehydrogenase (GDH) from *Pseudomonas sp*. was obtained from Sigma-Aldrich (Steinheim, Germany). *PfuS* DNA polymerase was produced in-house. The chemical 4-hydroxy-isophorone was synthesized and provided by InnoSyn B.V. (Geelen, Netherlands).

### Site-saturation mutagenesis at positions F87 and A328 of P450 BM3

Saturation mutagenesis libraries on P450 BM3 WT (vector backbone pALXtreme-1a) at positions F87 and A328 were generated using a modified QuikChange protocol for DNA amplification. SSM328 was generated by Müller *et. al*.^50^. Oligonucleotides with following sequences were used as forward primers for position F87 5′-CAGGAGACGGGTTANNKACAAGCTGGACGCATG-3′ and position A328 5′-GCTGCGCTTATGGCCAACTNNKCCTGCGTTTTCCCTATATG-3′ with the complementary sequences for the reverse primers at annealing temperatures of 60 °C and 55 °C (for 30 s), respectively. Template DNA was removed by digestion with 20 U *DpnI* overnight and subsequent purification with the NucleoSpin® Gel and PCR Clean-up kit (Macherey-Nagel, Düren, Germany) according to manufacturer´s guideline. Transformation of the libraries was performed using chemically competent *Escherichia coli* BL21-Gold (DE3) lacI^Q1^ cells^51^, which were plated on LB agar plates supplemented with 50 µg mL^−1^ kanamycin. A NucleoSpin® Plasmid kit (Macherey-Nagel) was used for plasmid purification of 3 mL overnight cultures from single clones and sequencing of the P450 BM3 gene was performed at GATC Biotec (Konstanz, Germany).

### Cultivation of P450 BM3 in 96-deep well plates

Master plates were prepared (150 µL LB media with 50 µg mL^−1^ kanamycin per well) in 96-well flat-bottom microtiter plates (MTP) by inoculation with either cell culture from glycerol stocks (5 µL) or single colonies from LB agar plates. Incubation was performed in a Multitron II shaker (Infors GmbH, Einsbach, Germany) for 16 h at 37 °C (900 rpm, 70% air humidity). Each master plate contained additionally 3–4 replicates of the negative (empty vector) and positive control (P450 BM3 wild type). For long-term storage of bacterial cells, glycerol was added to cell cultures in a final concentration of 25% (v/v). The resulting cryogenic cultures were stored at −80 °C. For protein expression, 6 µL cryo-culture were transferred to 96-deep-well plates (round bottom, 2.2 mL, Brand GmbH, Wertheim, Germany) containing 600 µL terrific broth (TB) media supplemented 1:1000 with kanamycin (50 mg mL^−1^), aminolevulinic acid (0.5 M), IPTG (0.1 M), thiamine (100 g L^−1^) and trace element solution (0.5 g L^−1^ CaCl_2_ × 2 H_2_O, 0.18 g L^−1^ ZnSO_4_ × 7 H_2_O, 0.1 g L^−1^ MnSO_4_ × H_2_O, 20.1 g L^−1^ EDTA-Na_2_ × 2 H_2_O, 16.7 g L^−1^ FeCl × 6 H_2_O, 0.16 g L^−1^ CuSO_4_ × 5 H_2_O, 0.18 g L^−1^ CoCl_2_ × 6 H_2_O). Cells were cultivated for 20 h at 30 °C (900 rpm, 70% air humidity) in a Multitron II MTP shaker. Expression plates were harvested by centrifugation (3220 × g, 15 min, 4 °C) and cell pellets stored at −20 °C until further use.

### Screening of P450 BM3 mutagenesis libraries in 96-well format

Frozen pellets of the microtiter-based cell cultures were thawed (10 min, ambient temperature) and 150 µL potassium phosphate (KPi) buffer (50 mM, pH 7.5) were added per well. Samples were incubated (5 min, ambient temperature), vortexed until all pellets were resuspended and supplemented with 150 µL lysozyme solution (5 mg mL^−1^ lysozyme in 50 mM KPi, pH 7.5) per well and incubated 1 h at 37 °C (900 rpm, 70% air humidity) in a MTP shaker. Of the disrupted cell cultures 250 µL per well were transferred to 96-well V-bottom microtiter plates and centrifuged (3220 × g, 15 min, 4 °C). The resulting lysates were used for screening experiments.

#### NADPH depletion measurements

Screening for increased NADPH oxidation rates was conducted in a Sunrise™ absorbance reader (Tecan Group Ltd., Männedorf, Switzerland) as described by Glieder and Meinhold (2003)^20^. MTPs (96-well flat-bottom) contained 30 µL lysate, 20 mM substrate, 2% (v/v) DMSO, 50 µL NADPH (240 µM final concentration) and KPi (50 mM, pH 7.5) in a total volume of 250 µL. Two identical setups were prepared with one containing the substrate (alpha-isophorone) and the other one serving as a reference without substrate (DMSO). MTPs were incubated for 5 min and the background absorbance measured at 340 nm before the reaction was started by addition of NADPH, followed by shaking for 2 s. The decrease in absorbance was measured at 340 nm every 10 s for 30 cycles.

#### Multiplexed capillary electrophoresis screening

For direct product profile analysis, libraries were screened via multiplexed capillary electrophoresis (MP-CE). Each MTP well contained 40 µL lysate, 20 mM substrate, 2% (v/v) DMSO, 50 µl NADPH regeneration mix (480 µM NADPH, 4 U mL^−1^ GDH, 60 mM glucose; final concentrations) and KPi (50 mM, pH 7.5) in a total volume of 250 µL. MTPs were sealed with a lid and incubated (900 rpm, 70% air humidity; 18 h at 25 °C) in a MTP shaker. Reaction was stopped by addition of 50 µL STOP mix (42 mM SDS in 15 mM sodium phosphate (NaPi), pH 7.45) supplemented with 0.78% (v/v) benzyl alcohol as internal standard. MTPs were shaken (400 rpm, 10 min), subsequently centrifuged (3220 × g, 20 min, 4 °C) and 100 µL supernatant of each well were transferred to a skirted 96-well PCR plate. Completed PCR plates were sealed with an adhesive film and stored at 4 °C until same-day use.

Capillary electrophoresis measurements were performed using a multiplexed cePRO 9600™ system operated with the pK_a_-Analyzer software (both supplied by Advanced Analytical Technologies, Ames, IA, USA). All experiments were conducted in a 96-capillary array with an inner diameter of 50 µm, an effective capillary length of 55 cm (80 cm total length). Air was cooled to 15 °C by a Peltier element and was channeled across the inlet of the capillary array. A voltage of 4.5 kV was applied with the anode on the inlet and the cathode on the outlet side, yielding an electric field of 56.25 V cm^−1^. Samples were injected hydrodynamically by applying a vacuum of −0.7 psi for ten seconds and UV detection of the samples was performed at 214 nm. The array was flushed between runs (70 psi, 5 min) with 0.1 M NaOH and deionized water, respectively, and then equilibrated with the electrophoresis buffer (30 mM SDS in 15 mM NaPi buffer, pH 7.45). For quantification, calibration curves of standards were generated from peak areas of each component.

### Expression and purification of P450 BM3 variants

Expression was performed in shaking flasks containing 20% (v/v) of TB media, supplemented 1:1000 (v/v) with kanamycin (50 mg mL^−1^) and trace element solution (0.5 g L^−1^ CaCl_2_ × 2 H_2_O, 0.18 g L^−1^ ZnSO_4_ × 7 H_2_O, 0.1 g L^−1^ MnSO_4_ × H_2_O, 20.1 g L^−1^ EDTA-Na_2_ × 2 H_2_O, 16.7 g L^−1^ FeCl × 6 H_2_O, 0.16 g L^−1^ CuSO_4_ × 5 H_2_O, 0.18 g L^−1^ CoCl_2_ × 6 H_2_O). After inoculation with a 1% (v/v) of a LB_kan_-overnight culture (*E. coli* BL21-Gold (DE3) lacI^Q^^1^ cells), cells were cultivated (37 °C, 250 rpm) to an OD_600_ of 0.8–1. Aminolevulinic acid (500 µM, final concentration) and thiamine (0.1 g L^−1^, final concentration) were added and expression induced by supplementing IPTG (100 µM; final concentration). Expression was continued at 30 °C for 20 h and cells harvested by centrifugation (3220 × g, 20 min, 4 °C).

For experiments with cell-free lysates, cell pellets of a 30 mL culture were resuspended in 3 mL 50 mM KPi, pH 7.5, homogenized by sonication for 4 min (30 s on +30 s off, 40% amplitude, Vibra-cell VCX-130, SONICS, Frankfurt, Germany) and centrifuged (13 000 × g, 30 min, 4 °C). The supernatant was transferred into fresh tubes and stored at 4 °C until further use.

For protein purification, frozen cell pellets from a 200 mL culture were resuspended in 15 mL 0.1 M Tris/HCl buffer (pH 7.8). Cells were homogenized by sonication for 4 min (30 s on + 30 s off, 40% amplitude, Vibra-cell VCX-130, SONICS) and subsequently disrupted in an Avestin EmulsiFlex-C3 high-pressure homogenizer (Ottawa, ON, Canada) by applying three cycles of 1500 bar pressure. After centrifugation (16 000 × g, 30 min, 4 °C) the supernatant was filtered through a 0.22 µm filter membrane. Purification of the P450 BM3 variants was performed as described elsewhere using a Toyopearl DEAE 650 S anion exchange matrix (Tosoh Bioscience, Stuttgart, Germany) and an ÄKTAprime Plus chromatography system with UV-detection (GE Healthcare, München, Germany)^52^. Elution of P450 BM3 variants was initiated using a salt gradient (1 M NaCl, 0.1 M Tris-HCl, pH 7.8) and fractions containing P450 BM3 enzyme were collected. Fractions were concentrated with an Amicon Ultra-4 centrifugation tube (50 kDa cut-off; Millipore, Schwalbach, Germany) and desalted using a PD-10 gel-filtration column (GE Healthcare, München, Germany) equilibrated with KPi (50 mM, pH 7.5). For long time storage, enzymes were shock-frozen in liquid nitrogen and lyophilized in a freeze-dryer (48 h, −54 °C, <0.01 mbar). Lyophilized enzyme samples were stored at −20 °C.

### Characterization of P450 BM3 variants

Concentrations of P450 BM3 variants were determined by recording CO difference spectroscopy following the protocol of Omura and Sato (1964)^53^.

#### Substrate conversion with cell-free extracts of screening variants

Product yields were obtained after conversion of alpha-isophorone with a GDH-glucose based NADPH cofactor regeneration system. Each sample contained: 1 μM P450 BM3 variant, 2.0 U mL^−1^ GDH, 360 mM glucose, 50 mM substrate, 2% DMSO (v/v), 500 μM NADPH in 1 mL KPi (50 mM, pH 7.5). Reactions were stirred in 3.5 mL screw cap vials with 400 rpm on multipoint magnetic stirrers (Variomag or Cimarec Poly 15, Thermo Scientific, Dreieich, Germany), quenched after 22 h with 100 µL 37% (v/v) HCl and used further for GC-FID analysis.

#### Characterization of P450 BM3 variants WT, F87V and A328N

Purified variants were characterized by their product profiles and determination of coupling efficiency and initial NADPH oxidation activity. Product profiles, including regioselectivity, yields and total turnover numbers were obtained after conversion of alpha-isophorone with a GDH-glucose based NADPH cofactor regeneration system. Each sample contained: 1 μM P450 BM3 variant, 3 U mL^−1^ GDH, 240 mM glucose, 20 mM substrate, 2% DMSO (v/v), 500 μM NADPH in 1 mL KPi (50 mM, pH 7.5). Reactions were stopped after 22 h of stirring (400 rpm, Variomag or Cimarec Poly 15, Thermo Scientific, Dreieich, Germany) with 100 µL 37% (v/v) HCl. Coupling efficiencies were determined after full depletion of NADPH using 2 µM P450 BM3, 10 mM substrate, 2% DMSO (v/v) and 1 mM NADPH in 1 mL KPi (50 mM, pH 7.5) incubated at 30 °C. Samples from conversion experiments and coupling efficiency determinations were subsequently analyzed by GC-FID. P450 BM3 concentrations of 1 µM WT, 1 µM F87V, 0.2 µM A328N were used for determination of the NADPH oxidation activity at 30 °C. The reactions contained 10 mM substrate, 2% DMSO (v/v) in KPi (50 mM; pH 7.5) in a final volume of 1 mL using semi-micro cuvettes. After 3 min incubation, 0.5 mM NADPH was supplemented and the oxidation of the cofactor followed at 340 nm in a spectrophotometer (Varian Cary 50 UV, Agilent, Ratingen, Germany). Rates were calculated using the extinction coefficient of 6,220 m^–1^ cm^–1^ for NADPH.

### Product analysis by gas chromatography

Samples from substrate conversion and coupling efficiency determination were analyzed and quantified via gas chromatography with flame ionization detection (GC-FID). Extraction was performed with ethyl acetate (1:1) as solvent supplemented with 2.6 µL mL^−1^ benzyl alcohol as internal standard and the resulting mixture was transferred in 2 mL safe lock tubes and vortexed (60–70% maximal force, 20 min; Vortex Genie 2, Scientific Industries, Bohemia, NY, USA). After centrifugation (11 000 × g, 5 min), the upper organic phase was transferred to new 2 mL tubes containing 50 mg water free sodium sulfate for drying. Samples were vortexed (60–70% maximal force, 5 min), centrifuged (11 000 × g, 5 min), and the dried organic phase was used for GC-FID measurements. Of each sample, 100 µL were filled in 1.5 mL glass screw neck vials equipped with 250 µL glass inlets. Analysis was performed in a GC-2010 gas chromatograph with auto sampler operated by the software GCsolution version 2.3 (Shimadzu Corp., Kyoto, Japan). A truncated 30 cm Optima^®^ 17-MS column from Macherey-Nagel (Düren, Germany) served for separation and following parameters were used: injection volume: 1 µL, injection temperature: 300 °C, injection mode: split, flow control mode: linear velocity, pressure: 114.9 kPa, total flow: 58.6 mL min^−1^, column flow: 2.65 mL min^−1^, linear velocity: 69.1 cm s^−1^, purge flow: 3.0 mL s^−1^, split ration: 20, temperature gradient: 100 °C, 5 °C min^−1^ to 175 °C, 20 °C min^−1^ to 250 °C, detection temperature: 300 °C, sampling rate: 40 msec, H_2_ flow: 40 mL min^−1^, makeup gas N_2_/air: 30 mL min^−1^ / 400 mL min^−1^. Concentrations of isophorone derivatives were calculated from corresponding calibration curves of standards.

## Supplementary information

Supplementary: A 96-multiplex capillary electrophoresis screening platform for product based evolution of P450 BM3

## Data Availability

The datasets generated during and/or analyzed during the current study are available from the corresponding author on reasonable request. The pALXtreme-1a plasmid and the strain *E. coli* BL21-Gold (DE3) lacI^Q1^ used in this study are available from the corresponding author upon reasonable request.
