# Supplementary material for: A 96-multiplex capillary electrophoresis screening platform for product based evolution of P450 BM3
Source: Sci Rep. 2019 Oct 29;9:15479. doi: 10.1038/s41598-019-52077-w (PMC6820799; doi:10.1038/s41598-019-52077-w)
Supplement: Supplementary file 1 — Supplementary: A 96-multiplex capillary electrophoresis screening platform for product based evolution of P450 BM3 [file 41598_2019_52077_MOESM1_ESM.pdf]

Supplementary: A 96-multiplex capillary electrophoresis screening platform for product based evolution of P450 BM3

[Authors: Anna Gärtner, Anna J. Ruff and Ulrich Schwaneberg]

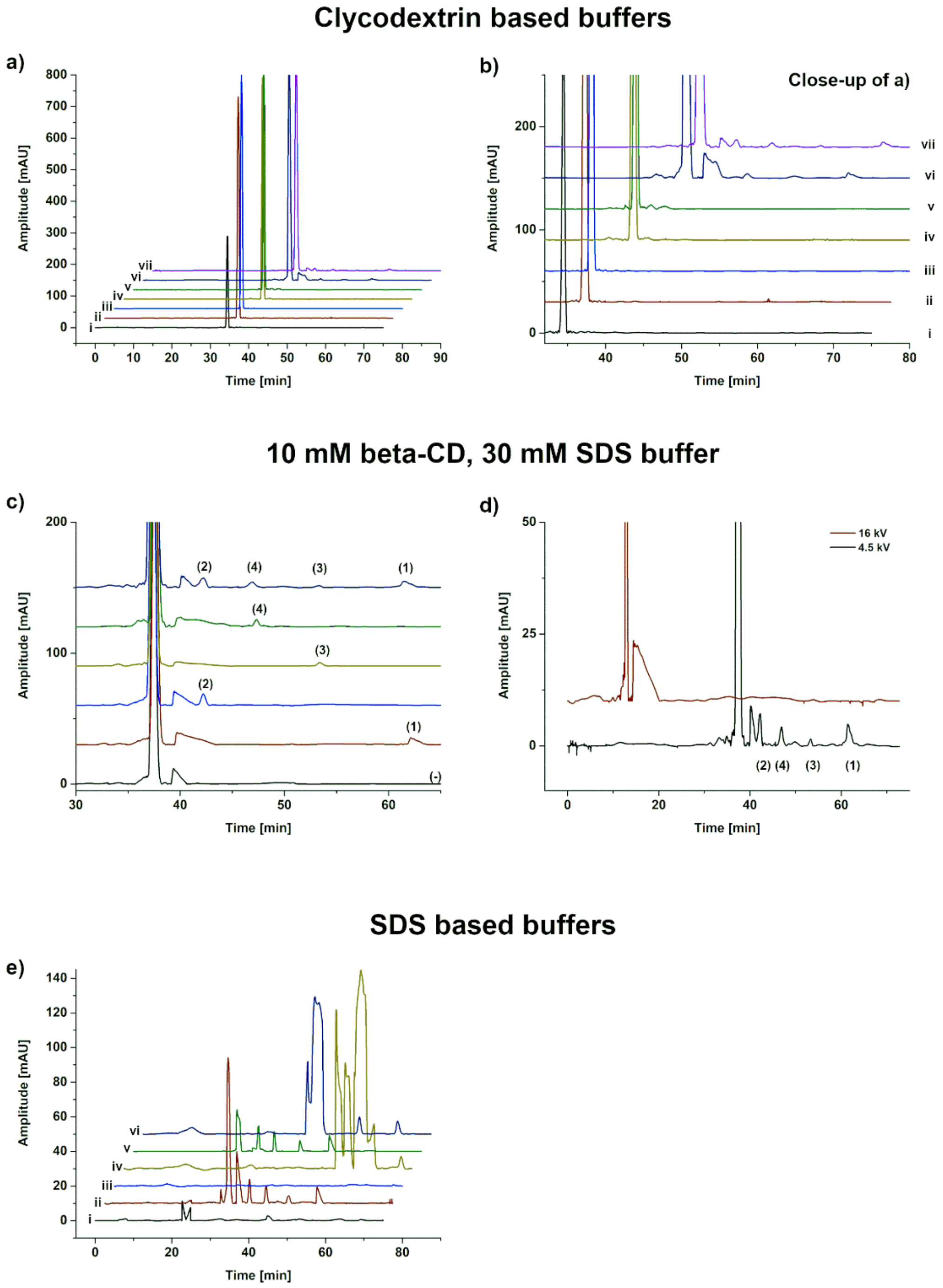

Figure S1 Electropherograms of isophorone derivate separation with different buffer additives. All additives were tested in 15 mM sodium phosphate (NaPi) buffer with pH 7.45. Isophorone derivates ((1) alpha-isophorone, (2) 4-hydroxy-isophorone, (3) isophorone oxide, (4) keto-isophorone) were

applied in a mixture (0.5 mM each derivate, pre-solved in DMSO or ethanol) if not stated otherwise. **a)** Cyclodextrine (CD) based buffers: i) no additive, ii) 20 mM alpha-CD, iii) 10 mM beta-CD, iv) 20 mM alpha-CD, 10 mM 18-crown-6, v) 10 mM beta-CD, 10 mM 18-crown-6, vi) 20 mM alpha-CD, 30 mM SDS, vii) 10 mM beta-CD, 30 mM SDS. Separation was performed at 4.5 kV. **b)** Close-up of **a)**. **c)** Separation of isophorone derivates in 10 mM beta-CD, 30 mM SDS buffer at 4.5 kV. **d)** Separation of isophorone derivate mixture in 10 mM beta-CD, 30 mM SDS buffer at different voltages. **e)** SDS based buffers: i) 10 mM beta-CD, 30 mM SDS, ii) 25 mM beta-CD, 50 mM SDS iii) 10 mM beta-CD, 30 mM SDS, 1 mM urea, iv) 25 mM beta-CD, 50 mM SDS, 1 mM urea, v) 30 mM SDS, vi) 50 mM SDS. Separation was performed at 4.5 kV.

Discussion Figure S1

Structural similarity and the uncharged nature of the isophorone derivates impede their electrophoretic separation with the MP-CE tool. Several additives like alpha- and beta-cyclodextrine (CD) and 18-crown-6 ether are reported to increase resolution of compounds with close structural similarity like enantiomers[1-3]. Addition of surfactants like SDS is applied in micellar electrokinetic chromatography (MEKC) for the separation of neutral molecules[4]. In the initial approach, 20 mM alpha-CD (Mw = 972.84 g/mol) or 10 mM beta-CD (Mw = 1134.98 g/mol) were solved 15 mM sodium phosphate (NaPi) buffer (pH 7.45) and separation analyzed with or without addition of 10 mM 18-crown-6 ether or 30 mM SDS (Figs S1a and b). Addition of SDS to both CDs seemed to allow separation of all derivates. However, with alpha-CD in the background 4-hydroxy-isophorone overlapped with a system peak which was not the case in presence of beta-CD in the background analyte (Fig. S1c). Increase of the separation voltage to 16 kV (upper limit of MP-CE device) in 10 mM beta-CD, 30 mM SDS buffer (Fig. S1d) prevented sufficient separation of isophorone derivates. Hence, further analysis was performed at 4.5 kV applying different concentrations of beta-CD and SDS in the background analyte (Fig. S1e). Further, urea was introduced as additive to allow solvation of higher beta-CD concentrations (25 mM)[5]. Increase of beta-CD and SDS concentration led to increased compound signals (Fig. S1 e) lane i vs. ii). Addition of urea was unfavorable for adequate separation in an appropriate time frame (lane iii vs. iv). Presence of SDS (30 mM) alone led to resolution of isophorone derivates (lane v). As separations is performed towards the cathode, separation was delayed upon increase of SDS concentration (lane vi).

In summary, supply of additives like alpha- and beta-CD, and 18-crown-6 ether, which are applied for separation of structurally similar compounds, was not necessary. Introduction of negative charge to the neutral compounds by addition of SDS was sufficient for derivate resolution.

Table S1 Peak resolution of MP-CE method at different separation voltages. Separation of isophorone derivates (alpha-isophorone (1), 4-hydroxy-isophorone (2), isophorone oxide (3), keto-isophorone (4)) and the internal standard benzyl alcohol (5) was performed in 30 mM SDS, 15 mM NaPi buffer (pH 7.45). Corresponding electropherograms are depicted in Fig. 2 in the Results section.

**Separation voltage**	**(2)-(5)**	**(5)-(4)**	**(4)-(3)**	**(3)-(1)**
**4.5 kV**	1.59 ± 0.04	4.15 ± 0.05	6.21 ± 0.09	4.00 ± 0.08
**8 kV**	1.11 ± 0.04	2.75 ± 0.07	5.40 ± 0.10	4.20 ± 0.17

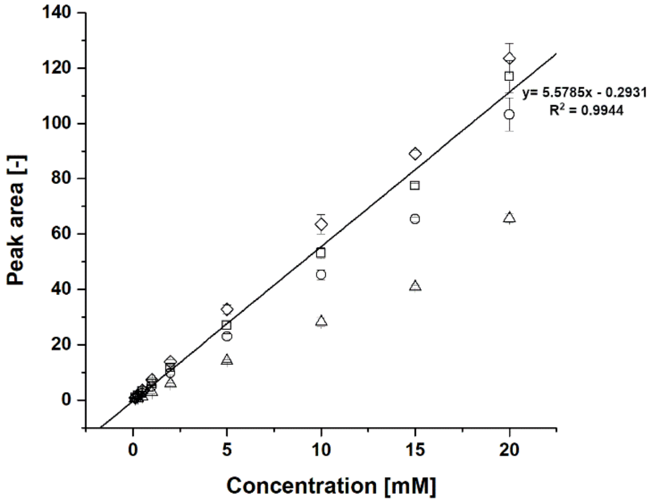

Figure S2 Standard curves of isophorone compounds in the MP-CE system. Isophorone derivates (alpha-isophorone: diamond, 4-hydroxy-isophorone: square, keto-isophorone: circle, isophorone oxide: triangle) showed a linear signal response (mean of 9 to 24 values ± s.e.) using a concentration range of 0.125-20 mM. The regression curve of the 4-hydroxy-isophorone data set is depicted as straight line.

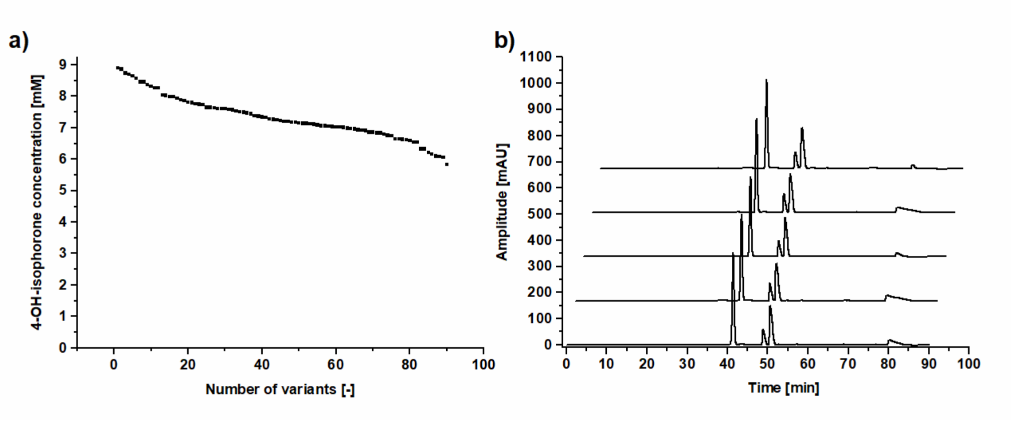

Figure S3 a) Deviation of 4-hydroxy-isophorone formation by P450 BM3 wild type within the CE screening system. From 96 capillaries of the CE head only 90 were usable (n = 5 biologically independent experiments). The mean of the individual relative standard deviations of each MTP measurement was calculated as 12.2% ± 1.8%. Shown above is the analysis of one measurement with a relative standard deviation of 9.3%. **b) Electropherograms of 5 wells containing P450 BM3 WT obtained from MP-CE measurements.**

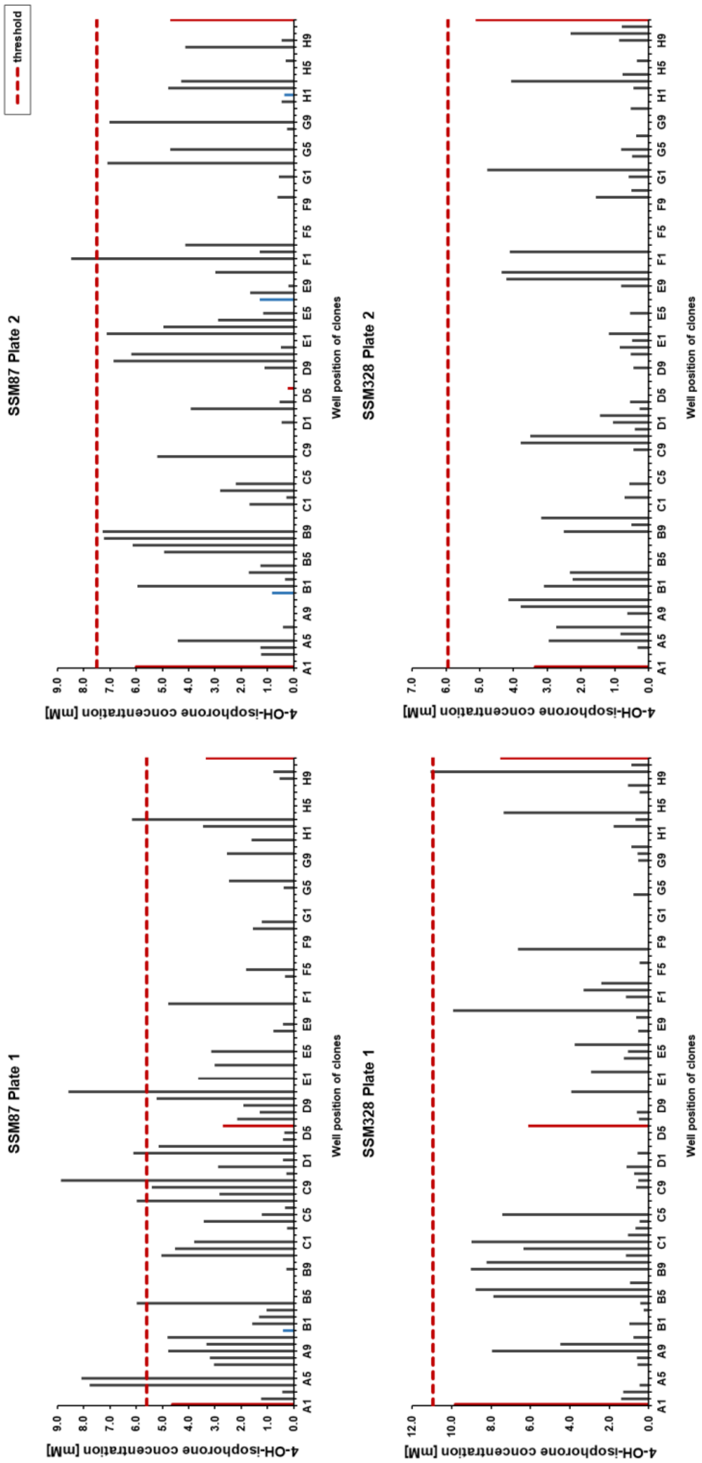

Figure S4 Results of MP-CE based screening of SSM87 and SSM328. Variants with an increase in 4-hydroxy-isophorone formation of factor ≥ 1.4 (dashed red line) compared to the mean of 2-3 WT values (red columns) were selected for a second screening round. Of each library, SSM87 and SSM328, 180 variants distributed in two 96-well MTP plates were analyzed. On each MTP plate, 3 wells contained EV as negative control (blue columns). False positive variants with unintentional mutations were excluded from rescreening.

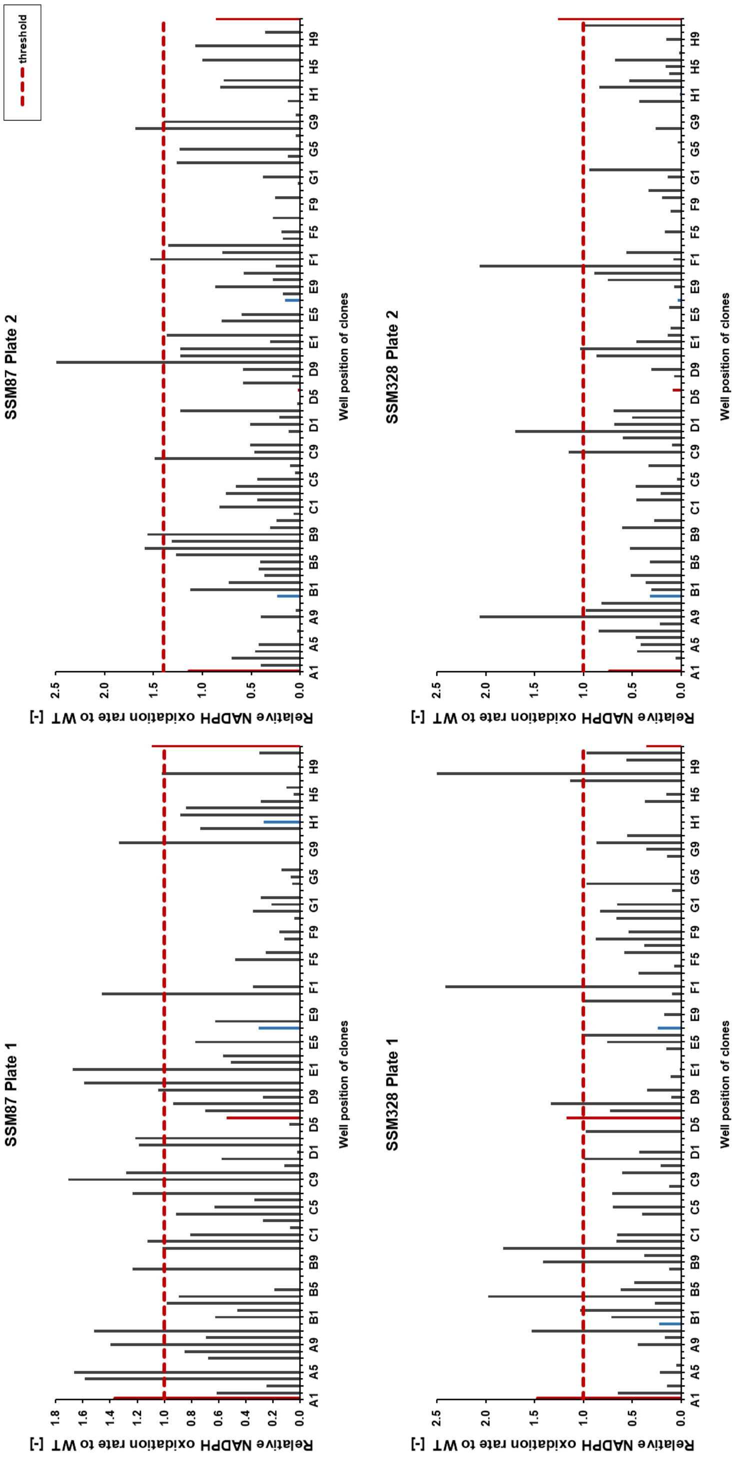

Figure S5 Results of NADPH depletion based screening of SSM87 and SSM328. Variants with an increase in NADPH oxidation consumption of factor ≥ 1.4 (dashed red line) compared to the mean of 2-3 WT values (red columns) were selected for a second screening round. Of each library, SSM87 and SSM328, 180 variants distributed in two 96-well MTP plates were analyzed. On each MTP plate, 3 wells contained EV as negative control (blue columns). False positive variants with unintentional mutations were excluded from rescreening.

Table S2 Relative performance of selected variants to WT. Variants that showed improvements in the respective screening platform were re-examined in triplicates in terms of their performance within the same screening platform. In bold, variants with 1.3-fold improvement (~2.5 x standard deviation) in NADPH consumption or 4-OH-isophorone formation compared to the WT (according to the selection method) were chosen for further characterization. For a full overview, chosen variants were also measured with the competing method. (mean ± s.e.; n = 4, biologically independent experiments)

**NADPH based selection**						
			**NADPH screening**		**CE screening**	
**Variant**		**Substitution**	**Mean**	**Deviation**	**Mean**	**Deviation**
**Controls**	WT	Phe87/Ala328	1.00	0.21	1.00	0.25
	EV		0.02	0.28	0.02	0.03
**SSM 87 P1**	**A4**	**Val**	**1.53**	0.49	1.48	0.52
	A5	Val	1.25	0.48	1.47	0.35
	A11	Phe	0.99	0.43	1.11	0.26
	**C9**	**Thr**	**2.03**	0.68	1.34	0.31
	**D11**	**Val**	**1.77**	0.34	1.71	0.36
	**E12**	**Val**	**1.50**	0.21	1.60	0.36
**SSM 87 P2**	B7	Phe	0.94	0.26	0.98	0.31
	**B9**	**Val**	**1.33**	0.47	1.11	0.24
	C8	Phe	0.92	0.40	0.99	0.37
	**E2**	**Phe**	**1.31**	0.31	1.08	0.29
	F1	Phe	0.91	0.28	0.98	0.20
	**G8**	**Pro**	**2.12**	0.44	0.19	0.09
**SSM328 P1**	**B4**	**Asn**	**3.21**	0.44	0.10	0.05
	**B11**	**Leu**	**2.53**	0.44	0.12	0.04
	**F1**	**Asn**	**3.29**	0.60	0.12	0.04
	**H8**	**Asn**	**3.00**	0.45	0.10	0.03
**SSM328 P2**	**A9**	**Asn**	**3.00**	0.54	0.09	0.04
	**C12**	**Asn**	**3.33**	0.36	0.09	0.04
	**E12**	**Asn**	**3.04**	0.56	0.10	0.04
**CE based selection**						
			**CE screening**		**NADPH screening**	
**Variant**		**Substitution**	**Mean**	**Deviation**	**Mean**	**Deviation**
**Controls**	WT	Phe87/Ala328	1.00	0.24	1.00	0.22
	EV		0.05	0.08	-0.01	0.18
**SSM 87 P1**	**A4**	**Val**	**1.48**	0.44	1.42	0.29
	**A5**	**Val**	**1.37**	0.37	1.58	0.17
	B4	Phe	0.92	0.23	1.00	0.22
	**C7**	**Val**	**1.61**	0.40	1.64	0.36
	**C10**	**Val**	**1.81**	0.38	2.02	0.27
	D2	Ala	1.02	0.34	1.27	0.29
	**D11**	**Val**	**1.63**	0.40	1.72	0.38
	H3	Phe	0.98	0.32	1.24	0.40
**SSM 87 P2**	F1	Phe	1.06	0.26	1.30	0.31
**SSM328 P1**	H10	Val	0.91	0.23	0.21	0.23

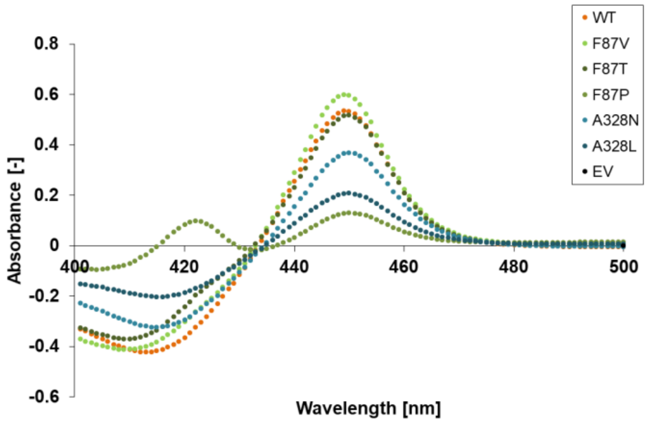

Figure S6 CO-difference spectra of selected variants. Enzyme concentrations and correct folding of P450s were determined by recording CO-difference spectra. Correctly folded P450 results in an absorbance maximum at 450 nm during measurements whilst incorrect folding leads to a maximum at ~420 nm.

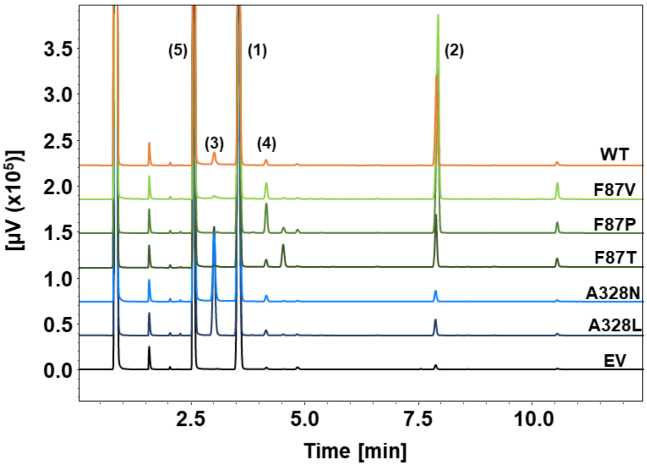

Figure S7 Product profiles of variants identified via CE and NADPH depletion based screening systems. Analysis of product formation after 24 h conversion of alpha-isophorone was performed by GC-FID. Variant F87V was identified by CE and NADPH depletion screening and variants F87T, F87P, A328N and A328L by NADPH depletion screening. Conversion were performed with normalized P450 content using lysates and a NADPH regeneration system. Lysate of cells expressing the empty vector (EV) not carrying a P450 BM3 gene was taken as negative control. Retention time: < 2.5 min solvents, 2.6 min internal standard benzyl alcohol (5), 3.1 min isophorone oxide (3), 3.6 min alpha-isophorone (1), 4.2 min keto-isophorone (4), 7.9 min 4-hydroxy-isophorone (2), remaining small peaks > 4.5 min unidentified side products.

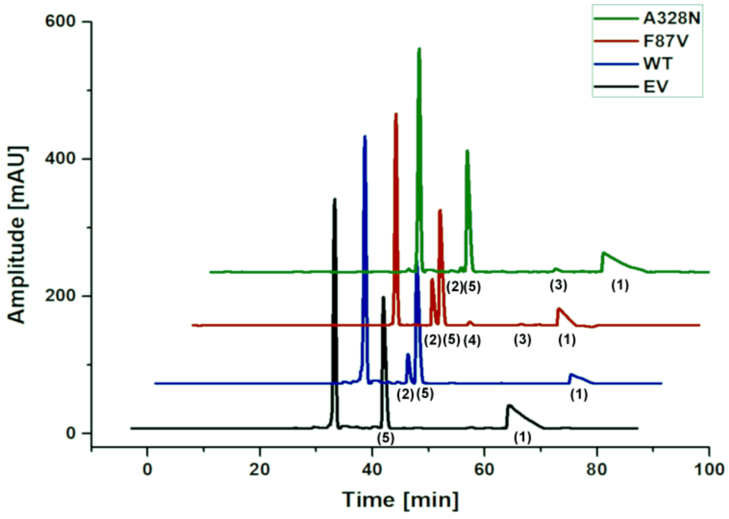

Figure S8 MP-CE electropherograms of variants F87V and A328N compared to WT and EV. Electropherograms were obtained during first round of screening of SSM libraries (two separate measurements) detecting following compounds: Alpha-isophorone (1), 4-hydroxy-isophorone (2), isophorone oxide (3), keto-isophorone (4) and the internal standard benzyl alcohol (5).

Table S3 Comparison of different screening tools towards their key factors. Sample preparation was not considered for comparison. Data for HPLC separation was taken from Kaluzna *et al.*[6].

Key factor	Parameter	NADPH depletion assay	MP-CE	HPLC
**Time**	Samples per run	96	96	1
	Analysis time per run	11 min	40-60 min	25 min
	Analysis time per sample	6.9 s	25-37.5 s	25 min
**Costs**	Solvent volume per sample	0 mL	1.875 mL	37.5 mL
**Quality**	Informative value	low	very high	very high
	Number of false positives	high	low	very low

Discussion Table S3

For the evaluation of successful high-throughput screening (HTS) systems time, costs and quality form the key factors.[7] All three factors are closely connected and changes in the setup of one factor will have influence on other factors. In the following, the screening tools analyzed within the manuscript (NADPH depletion and MP-CE) are compared to each other with respect to the named key factors. In addition, both latter tools are compared to a HPLC method established by Kaluzna *et al.* for analysis of alpha-isophorone turnover by P450 BM3 WT.[6] Comparison is focused on parameters during measurement

itself. The Table S2 summarizes the key values for the three tools. The NADPH depletion and MP-CE tool both allow low analysis time per sample (in range of seconds) as 96 samples can be measured simultaneously while several minutes of analysis time are necessary for one sample when using HPLC. As absorbance measurement during NADPH depletion assay does not demand extra reagents, analysis costs for reagents are theoretically not existent. For separation with MP-CE and HPLC liquid background solvents are needed. Due to the thin diameter of the MP-CE capillaries, little solvent volume (1.875 mL for washing and separation) is needed per sample. For one HPLC run 37.5 mL solvent are needed which increases the total reagents costs per analyzed sample. However, regarding the quality of the informative value MP-CE and HPLC perform superior to NADPH depletion assay. Target product formation is not directly coupled to NADPH consumption due to uncoupling effects and side product formation of P450 BM3. Direct read out of several products with the MP-CE and HPLC tool allow accurate selection of variants. When working with volatile compounds, MP-CE has a slight disadvantage over HPLC as MP-CE samples are introduced unsealed into the device. In summary, the MP-CE measurement combines the benefits of NADPH depletion and HPLC and allows fast analysis under low regent costs with high analysis quality.

References

1 Fanali, S. Enantioselective determination by capillary electrophoresis with cyclodextrins as chiral selectors. *J. Chromatogr. A* **875**, 89-122, doi:10.1016/S0021-9673(99)01309-6 (2000).

2 Huang, W. X., Fazio, S. D. & Vivilecchia, R. V. Achievement of enantioselectivity of non-polar primary amines by a non-chiral crown ether. *J. Chromatogr. A* **781**, 129-137, doi:10.1016/S0021-9673(97)00541-4 (1997).

3 Nishi, H., Fukuyama, T. & Terabe, S. Chiral Separation by Cyclodextrin-Modified Micellar Electrokinetic Chromatography. *J. Chromatogr.* **553**, 503-516, doi:10.1016/S0021-9673(01)88522-8 (1991).

4 Hancu, G., Simon, B., Rusu, A., Mircia, E. & Gyéresi, Á. Principles of Micellar Electrokinetic Capillary Chromatography Applied in Pharmaceutical Analysis. *Adv. Pharm. Bull.* **3**, 1-8, doi:10.5681/apb.2013.001 (2013).

5 Hedges, A. in *Starch Chemistry and Technology* Ch. 22, 833-851 (Academic Press, 2009).

6 Kaluzna, I. *et al.* Enabling Selective and Sustainable P450 Oxygenation Technology. Production of 4-Hydroxy-α-isophorone on Kilogram Scale. *Org. Process Res. Dev.* **20**, 814−819, doi:10.1021/acs.oprd.5b0028210.1021/acs.oprd.5b00282 (2016).

7 Mayr, L. M. & Fuerst, P. The Future of High-Throughput Screening. *J. Biomol. Screen.* **13**, 443-448, doi:10.1177/1087057108319644 (2008).
